# Airborne Alt a 1 Dynamic and Its Relationship with the Airborne Dynamics of *Alternaria* Conidia and *Pleosporales* Spores

**DOI:** 10.3390/jof8020125

**Published:** 2022-01-27

**Authors:** Concepción De Linares, David Navarro, Rut Puigdemunt, Jordina Belmonte

**Affiliations:** 1 Department of Botany, University of Granada, 180171 Granada, Spain; 2 Institut de Ciència i Tecnologia Ambientals (ICTA-UAB), Universitat Autònoma de Barcelona (UAB), 08193 Bellaterra, Spain; david.navarro@uab.cat (D.N.); rut.puigdemunt@uab.cat (R.P.); jordina.belmonte@uab.cat (J.B.); 3 Departament de Biologia Animal, Biologia Vegetal i Ecologia, Universitat Autònoma de Barcelona (UAB), 08193 Bellaterra, Spain

**Keywords:** allergens, Alt a 1, *Alternaria*, *Pleosporales*, fungal spores, aerobiological samplers

## Abstract

Fungal spores are universal atmospheric components associated to allergic reactions. *Alternaria* (Ascomycota) is considered the most allergenic spore taxa. Alt a 1 is the major allergen of *Alternaria* and is present also in other *Pleosporales*. In this study, standard Hirst-based sampling and analyzing methods for measuring spore daily concentrations of *Alternaria*, *Curvularia*, *Drechslera-Helminthosporium*, *Epicoccum*, *Leptosphaeria*, *Pithomyces*, *Pleospora* and *Stemphylium* (all included in the taxon *Pleosporales*) have been used as well as two high-volume samplers, Burkard Cyclone (2017) and MCV CAV-A/mb (2019–2020), and ELISA kits for measuring the allergen. The detection and quantification of Alt a 1 was only possible in the samples from the MCV sampler. Although Alt a 1 was better correlated with *Alternaria* spores than with *Pleosporales* spores, the three of them showed high correlations. It is shown that there is a high and significant correlation of Alt a 1 with temperature, a negative correlation with relative humidity and no correlation with precipitation. The aerobiological monitoring of these three elements ensures the best information for understanding the affectation to allergy sufferers, but, if this is not possible, as a minimum public health service aimed at the detection, treatment and prevention of allergies, the study of the airborne *Alternaria* spores should be ensured.

## 1. Introduction

Fungal spores are universal, atmospheric indoor and outdoor components. Aerobiological studies have shown most fungal spores in outdoor air to be from the phyla Ascomycota and Basidiomycota [1,2]. Airborne spores are also referred to as ascospores or basidiospores when they result from a sexual reproduction process and as conidia when they are asexually produced [3]. According to Kołodziejczyk et al. [4], allergic reactions associated with fungi involving the lower respiratory tract are more severe than other types of allergies. More than 80 genera of fungi have been associated with symptoms of lower respiratory tract allergy [5]. *Alternaria* Nees is a ubiquitous fungal genus that includes saprobic, endophytic and pathogenic species associated with a wide variety of substrates such as seeds, plants, agricultural products, animals, soil and the atmosphere [6].

*Alternaria* (Ascomycota, Cl. Dothideomycetes) produces asexual reproductive wind-dispersed conidia that are one of the most frequently encountered in the outdoor environment and are considered as the most allergenic fungal spores [7]. Its role in allergies was first recognized by Hopkins et al. [8]. *Alternaria* conidia are related to respiratory allergic diseases through the activation of IgE-mediated antibodies producing rhinitis, asthma and atopic dermatitis [9,10,11]. Up to now, the exact prevalence of *Alternaria* allergens has not been established since reports of skin prick test (SPT) reactivity to this fungus range between 3 and 91%, depending upon the population studied, extracts used and species tested [5,12,13]. In Spain [14], in a population of 1156 patients, 18.4% and 14.1% SPTs resulted as positive to *Alternaria alternata* (Fr.) Keissl. and Alt a 1, respectively, and 92% suffered from allergic rhinitis. The same authors showed results by regions and, in the study area (Catalonia), the percentage of positive IgE to *A. alternata* and Alt a 1 was 17.8% with children more affected than adults (7.7%). *Alternaria* conidia also have clinical significance for producing toxic secondary metabolites involved in cutaneous, osteomyelitis, pulmonary infections and keratomycosis in humans [15], cancer in mammals [16], phaeohyphomycotic infection in humans [6], cats [17] and horses [18] and mycotoxicosis in farms [19], among others. As a plant pathogen and ubiquitous saprophyte [20], *Alternaria* causes serious impacts (including economic impacts) on a large variety of crops, i.e., olive, small grain cereals, tobacco, cauliflower, broccoli, pepper, carrot, potato and fruits such as tomato, citrus, melon and apple [16,21].

Due to all these disturbances to human interests, *Alternaria* conidia in the air have been studied considerably [22,23,24,25].

There are currently sixteen known allergens of *A. alternata* [26], twelve of them listed in the official database of the WHO/IUIS Subcommittee on Allergen Nomenclature. The major allergen, and highly allergenic protein, is Alt a 1, identified as a protein formed by a two-chain dimer linked by disulfide bonds with a molecular weight of approximately 30 kDa [27]. Despite more than two decades of studies on Alt a 1, its sequence is recognized only in part, and its biological function remains unknown [28]. Although Alt a 1 is found in the cell wall of *Alternaria* spores [26], it is secreted or released from growing germ tubes emerging from the spores [29]. Green et al. [30,31] showed the important contribution of airborne hyphae and germinated conidia (more than ungerminated ones) to the presence of allergens in the environment.

Since the end of the 1990s, some aerobiologists have broadened the focus of their research to molecular aerobiology [32,33,34,35,36,37]. Knowledge of the dynamics of not only the airborne concentrations of pollen/spores but also of their aeroallergens contributes to a better understanding of the cycles of symptoms of patients allergic to pollen/spores. Up to now, these studies have focused on pollen allergens, being the fungal aeroallergens that are less studied.

As Alt a 1 is present in a great number of taxa including the Dothideomycetes O.E. Erikss. & Winka and Sordariomycetes O.E. Erikss. & Winka, especially in the order Pleosporales Luttr. ex M.E. Barr [38,39,40,41,42,43]. To better understand the relationship between the airborne concentrations of *Alternaria* conidia and the allergen Alt a 1 and to establish the period with allergy risk to Alt a 1, we must also consider other taxa belonging to the *Pleosporaceae Nitschke* family [1,41,42,44], and the Pleosporales order [45].

The aim of this paper was to detect and quantify the Alt a 1 allergen in the atmosphere of Bellaterra (Barcelona, NE Spain), and to compare its dynamics with those of *Alternaria* conidia and of the spores (conidia and ascospores) of the taxon *Pleosporales* identified in the airborne spectra containing Alt a 1 or homologous/orthologous allergens. The taxon *Pleosporales* was then constituted, together with *Alternaria*, by the spores of *Curvularia* Boedijn [38,39]; *Drechslera* S. Ito-*Helminthosporium* Link [7]; *Epicoccum* Link [46]; *Leptosphaeria* Ces. & De Not. [43]; *Pithomyces* Berk. & Broome [31]; *Pleospora* Rabenh. ex Ces. & De Not. [7,43]; *Stemphylium* Wallr. [38,39,41,44].

The comparison between the traditional aerobiological study and the allergenic one was expected to show up the best method to establish the period of respiratory allergy risk due to Alt a 1.

## 2. Materials and Methods

### 2.1. Study Area

The sampling station was located in the Universitat Autònoma de Barcelona, (Bellaterra, Barcelona, NE Spain), on the rooftop of the C building, at 23 m.a.g.l. (41°30′20″ N, 02°06′28″ E and 245 m.a.s.l.). Following Allue Andrade [47], the climate of this locality can be described as fresh (mean annual temperature 12.0–15.5 °C) and humid (total annual precipitation 400–700 mm) and the corresponding phytoclimate is Fresh-Continental Oriental-semihumid [3]. Under a more global climate classification system such as that of Köppen-Geiger updated by Peel et al. [48] and with data from the period 1981–2010, the region corresponds to Csa, defined as temperate with dry and hot summer [49]. Regarding the plant landscape, the area is included in the Mediterranean biogeographical region [24].

### 2.2. Sampling of Airborne Fungal Spores

The Aerobiological Network of Catalonia (Xarxa Aerobiològica de Catalunya, XAC, https://aerobiologia.cat, accessed on 26 November 2021) has been studying the daily airborne fungal spores at the site of Bellaterra (Barcelona) uninterruptedly since 1995. The sampling instrument is a 7-day recorder VPPS 2000 spore trap (Lanzoni S.r.l., Bologna, Italy), based on Hirst [50], as recommended by the Spanish Aerobiological Network (Red Española de Aerobiología, REA, Galán et al. [51] and at international level [52]. Hirst’s collector was designed specifically for the intake of pollen, spores and other particles suspended in the air, at an aspiration flow rate of 10 L/min, comparable with the respiration of an average adult human. It is a continuous volumetric sampler with wind orientation of the intake orifice, and, thanks to an aspiration system, the collected particles are deposited on a plastic band coated with silicone oil tightly deposited around a drum that is displaced at a rate of 2 mm/h by means of a clockwise mechanism. Once a week the plastic band in the drum is replaced and the segments corresponding to each day (0–24 h UTC) mounted on a slide that is analyzed at the light microscope.

The counting method was that recommended by the REA [51] and following the minimum requirements of the European Aerobiology Society, EAS [52]. Terminology used in this paper follows the International Association for the Aerobiology (IAA) and EAS recommendations [53]. The data used in this study were expressed as daily average of spores per cubic meter of air (spores/m^3^) and Annual Spores Integral (ASIn), or addition of the average daily spores’ concentration over the year. For this study, the fungal taxa considered were *Alternaria* and *Pleosporales* (made up by the sum of the conidial genus *Curvularia*, *Drechslera-Helminthosporium*, *Epicoccum*, *Pithomyces* and *Stemphylium*, and the meiosporic ascosporal genus *Lepthosphaeria* and *Pleospora*) for the years 2017, 2019 and 2020.

### 2.3. Sampling of Airborne Allergens

The study of Alt a 1 was performed using two types of collectors and one analyzing method. On the one hand, in year 2017, we used a Burkard Cyclone sampler (Burkard Manufacturing Co. Ltd., Hertfordshire, England). This trap is a continuous volumetric sampler with wind orientation of the intake orifice, which captures solid particles from the air creating a cyclone effect, depositing said particles at the bottom of a 1.5-mL Eppendorf vial. The aspiration flow rate is 16.5 L/min. The collector provides a sample (Eppendorf) per day (0–24 h), thanks to an internal rotating mechanism that displaces from one to another in a set of 8 Eppendorf’s that are renovelled once a week. Based on Burkard manufacturing company, this collector offers 100% efficiency sampling down to a 1.06-µm range, decreasing to 93.82% for the size range 0.82 to 0.75 µm. Airborne samples were stored at −20 °C until their analysis. Before the analysis, the samples were hydrated for 6 h using 100 µL PBS solution.

On the other hand, in years 2019 and 2020, we used a high-volume sampler (MCV CAV-A/mb, ©MCV), designed in principle for the sampling of atmospheric particulate matter. It is a volumetric sampler, working at a flow intake rate of 400 L/min. and impelling the air against a fiberglass filter (UNE-EN12341:2015; UNE-EN 14907:2006a, b) that will retain the particles. Based on MCV S.L. Company, this collector complies with the specifications for High Volume Collectors contained in the UNE-EN 12341 and UNE-EN 14907 Standards. The total diameter of the filter is 15 cm, but the diameter of the impacted area is 12 cm. The filters are replaced manually. Therefore, as we were interested in daily measures (from 0 to 24 h), the sampling was carried out on alternate days. The samples were stored at 2 °C until their analysis. Before the analysis, a representative part of the daily filters, consisting of 8 circles of 0.5 cm in diameter obtained in selected areas, was equally distributed in two Eppendorf vials with 500 µL PBS solution and hydrated for 6 h [54].

Once hydrated, the samples obtained from the Burkard cyclone and the MCV high volume traps were analyzed according to the commercial ELISA kits for the detection of Alt a 1 (Indoor Biotechnologies, Inc., Cardiff, United Kingdom) according to the protocol provided on the supplier’s web site (Indoor Biotechnologies [55]). The allergen standards used were provided with the kits. The microplates used were NUNC™ microplates (Nunc, Rochester, NY, USA). Absorbance measurements were made at 450 µm filter in a Plate reader model Halo MpR-96 from Dynamica^®^ (DKSH, North Shore, New Zealand)

The results are expressed as daily allergen concentrations in nanograms of allergen per cubic meter of air (ng/m^3^) [51] and as Annual Allergen Integral (AAIn) obtained by summing the average daily allergen concentration over the year.

### 2.4. Meteorological Data

The meteorological variables considered in this study were maximum and minimum daily temperatures (expressed in Celsius degrees), daily rainfall (expressed in millimeters) and relative humidity (expressed in percentages). Data were obtained from the Servei Meteorològic de Catalunya and correspond to the Sabadell meteorological station.

### 2.5. Statistical Analysis

Spearman’s correlation coefficients between the daily concentrations of *Alternaria* conidia and *Pleosporales* spores (conidia + ascospores) and Alt a 1 allergen and daily meteorological variables were calculated. The analyses were carried out by using the IBM SPSS Statistic version 26.0 (IBM, Corp., Armonk, NY, USA).

## 3. Results

### 3.1. Summary of Aerobiological and Meteorological Results

Table 1 shows the summary (annual values) of the aerobiological parameters studied for Alt a 1, *Alternaria* spores (or conidia) and *Pleosporales* spores and for meteorological data. As shown, Alt a 1 was not detected with the Multi/vial cyclone sampler (N.D. not detected) in year 2017, although spores of *Alternaria* and *Pleosporales* were detected at levels similar to years 2019 and 2020. On the contrary, samples obtained with the high-volume device showed that the detection and quantification of Alt a 1 were possible to perform.

### 3.2. Airborne Fungal Aeroallergens vs. Spores and vs. Meteorology

The years studied showed differences between all parameters analyzed (Table 1). In 2017, Alt a 1 was not detected. In 2019, the Allergen Annual Integral (AAIn) was quantified in 861.5 ng·day/m^3^ of Alt a 1 in 155 out of 182 analyzed days, while in 2020 this value accounted for 1352.0 ng·day/m^3^ during 173 out of 183 analyzed days. In the case of peak allergen days, while the highest concentration was detected in 2020 (25 September), reaching 109.7 ng/m^3^, in 2019, it occurred on October 29 with 30.6 ng/m^3^. The Annual Spore Integral (ASIn) for the two fungal spore types studied showed similar results, although in year 2019, concentrations were lower than in year 2017 and year 2020. In each of the three years, peak days for *Alternaria* and *Pleosporales* coincided, showing the important contribution of *Alternaria* to *Pleosporales* (63.5%, 64.3% and 66.6% respectively). The date of the yearly peak concentration occurred always in autumn but in different months. In the year 2020, the peak of allergen concentration coincided with the peak of each spore taxon concentration. In relation to the meteorological parameters, they were similar in the three years for temperature, 2020 presented the highest relative humidity and much higher precipitation than 2017 and 2019.

**Figure 1:**
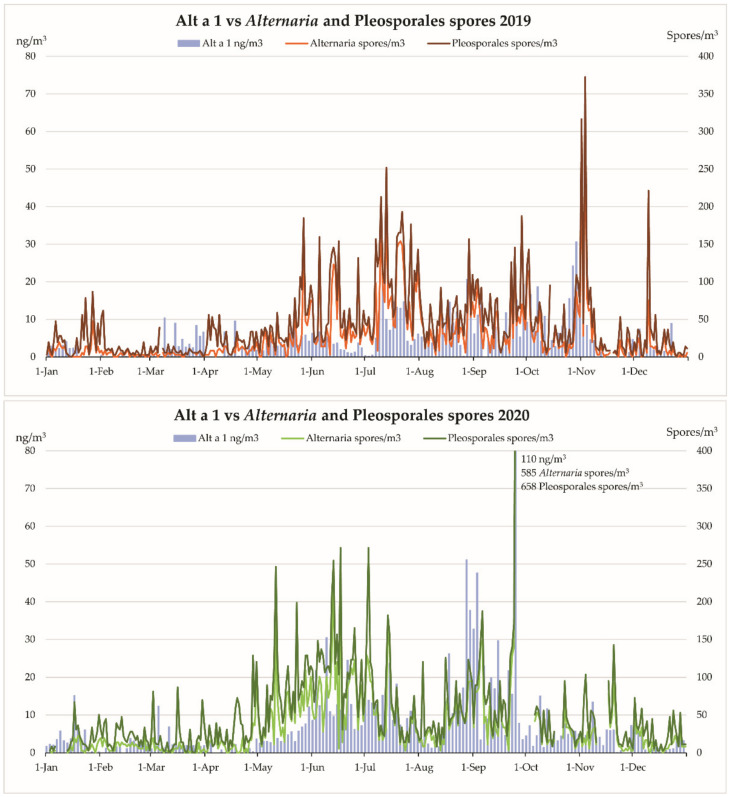
Comparison of the dynamics of Alt a 1 with *Alternaria* and *Pleosporales* spores in 2019 and 2020.

Figure 1 shows the annual patterns during the studied period of airborne Alt a 1, *Alternaria* and *Pleosporales* spores in 2019 and 2020. Both fungal spore types were present in the air throughout the year showing their maximum sporulation in the summer and autumn. In relation to the Alt a 1 aeroallergen dynamics, it was characterized by its continuous presence (alternate days) in 2019 and 2020, although with abundant oscillations. In both years, when *Alternaria* and *Pleosporales* spores registered the highest concentrations, the dynamics of the aeroallergen were similar. On the other hand, on 25 September 2020, the three variables increased significantly, reaching their maximum: Alt a 1: 109.7 ng/m^3^, *Alternaria*: 585 spores/m^3^ and *Pleosporales*: 658 spores/m^3^.

Results of the Spearman correlation test are shown in Table 2. Significant correlations (*p* < 0.01) between *Alternaria* and *Pleosporales* spores were observed for the three years, with 2019 showing the highest values and 2020 the lowest, although still important (0.856). Significant correlations (*p* < 0.01) between Alt a 1, *Alternaria* and *Pleosporales* spores during 2019 and 2020 were also registered, with higher values in 2020. The meteorological variables maximum, minimum and mean temperature (Tmax., Tmin., Tmean) showed positive and significative correlations (*p* < 0.01) with the biological airborne particles, while correlations with precipitation were negative (*p* < 0.01) for *Alternaria* spores (except in 2019), positive (*p* < 0.05) for *Pleosporales* spores (except in 2017) and not significant for Alt a 1. Correlations with relative humidity were negative in all cases, at *p* < 0.01 for *Alternaria* spores, and at *p* < 0.05 for *Pleosporales* spores (except in 2017) and Alt a 1 in year 2020.

## 4. Discussion

In the last decades, allergy diagnosis has changed, incorporating molecular analytical methods. In the same sense, aerobiology has evolved, including the detection and quantification of the allergenic load in the atmosphere by means of immunological technics as well as the traditional sampling and counting of the airborne pollen and spore particles. According to Cecchi [56], molecular aerobiology now represents the most intriguing achievement of research on allergenic pollen and could help to improve the assessment of pollen exposure, and research on epidemiology and clinical trials should include the results from standard pollen counts and allergen measurements. The present study offers an analysis of the airborne allergenic load of Alt a 1 in the environments of Bellaterra (Barcelona) in parallel to the study of the airborne fungal spores known to contain this allergen.

As already investigated by several authors for pollen [34,37,57,58,59,60] and in fewer studies for fungal spores [61,62,63], this paper is focused on the comparison of the daily concentrations of the allergenic molecule (Alt a 1) and the corresponding particles (spore taxa *Alternaria* and *Pleosporales*) as well as with the meteorological parameters.

A first step in this study was related to finding a sampling device able to provide enough samples to detect and quantify the aeroallergen Alt a 1 because it could not be measured in samples obtained in 2017 with a Multi/vial Cyclone sampler (aspiration rate of 16 L/min). Cyclone samplers were of common use and allowed to detect and quantify pollen allergens [57,58,64,65] but with the important limitation of measuring only one allergen type per daily sample [66]. The authors decided to use a high-volume sampler and, concretely, an MCV CAV-A/mb (with a suction capacity of 400 L/min). The choice of this sampler was based on Querol et al. [67], stating that it is one of the main standardized methods to control the total suspended particles (TPS) in the atmosphere, and with the hypothesis that it could be able to collect biological particles such as allergens at the same time as inorganic particles. This study has proven its utility to sample air with the aim to detect and quantify Alt a 1 through ELISA analyses. It has been proven also that more than one allergen per sample can be measured [54]. The only limitation the authors found to this sampler is that, wanting results from 0:00 to 23:59 h, samples were obtained for alternate days. To obtain samples for each day using this methodology we propose the use of two samplers, one alongside the other and sampling on alternate days.

In this study, *Alternaria* and *Pleosporales* spores were present throughout the year, with the main body of the sporulation lasting from May to November, the highest concentrations being recorded between the summer and autumn and data showing interannual variability (Table 1, Figure 1). These behaviors coincide with the dynamics revealed by these spore types in most studies [24,35,62]. The highest correlations found in this study corresponded to the daily concentrations of *Alternaria* and *Pleosporales spores*, varying between 0.875 (year 2019) to 0.856 (year 2020), with *p* < 0.01. Such high correlations could be expected, as *Alternaria* spores are the main components of the taxon *Pleosporales* (63.5% in 2017, 64.3% in 2019 and 66.7% in 2020). Although the highest correlation between the daily concentrations of *Alternaria* and *Pleosporales* spores was observed in the year 2019 (0.875), the highest correlations between the allergen Alt a 1 and *Alternaria* (0.631) and *Pleosporales* (0.556) spores were observed in the year 2020, the year showing the highest Annual Integrals for each one of the three airborne components under study. The explanation to this result could be the existence of variability in the allergenic potency of these spores through time as already shown by Grewling et al. [63] for *Alternaria* and for several pollen types and the corresponding allergens [56].

Brito et al. [62] is the only publication found to compare our results with. These authors ran a similar study in Ciudad Real (central Spain), in the year 2004. They adapted an Air Sentinel high-volume (10 m^3^/h) sampler to the sampling outdoor and measured Alt a 1 with ELISA methodology; at the same time they measured *Alternaria* spores with the same standard methodology used by the authors. Their results were comparable to those in this study for the year 2020 (*Alternaria* spores ASIn 10.476 Spore·day/m^3^, peak 626 spores/m^3^ on 8 June; Alt a 1 peak 110.25 pg/m^3^ on 16 November). Differences observed can be attributed to the variability already mentioned in the allergen content per spore [53,68,69] and to the meteorological and environmental conditions that influence the spore content in the air [5,24,25].

The summary of the results presented in Table 1 could lead to the deduction that lower maximum temperatures and higher minimum temperatures, rain, and relative humidity propagate higher concentrations of *Alternaria* and *Pleosporales* spores as well as of Alt a 1 allergen, but the correlation study of the daily values of these data (Table 2) shows that it is not so simple.

Correlation results showed that daily temperature (maximum, minimum and mean) was positively and significatively correlated with *Alternaria* and *Pleosporales spores*’ concentrations, as already stated for *Alternaria* by De Linares et al. [35]; Grinn-Gofroń and Bosiacka [70]; Damialis et al. [71]; Sousa et al. [72]; Vélez-Pereira et al. [25]. The same correlation was observed with Alt a 1, and this is (as far as we know) shown for the first time. Precipitation correlated significantly and negatively with *Alternaria* (except in 2019), positively with *Pleosporales* (except in 2017) and never correlated with Alt a 1. The results for *Alternaria* agree with those of De Linares et al. [35] and Vélez-Pereira et al. [25], and the complex relationship of different spore taxa (conidiospores, ascospores and basidiospores) with rainfall is well known [5,24] and explains the change of sign of the correlation with rainfall shown by the complex taxon Pleosporales. Relative humidity showed a significant negative correlation with both fungal spores’ taxa, coinciding with the results obtained by Maya-Manzano et al. [23] and Grinn-Gofroń et al. [24], and also with Alt a 1 in the year 2020, which is contrary to the results obtained by Brito et al. [62].

To show how difficult it is to establish the relationship between meteorological parameters and atmospheric biological particles, we analysed the meteorological conditions for the *Alternaria*, *Pleosporales* and Alt a 1 peak date in 2020 (25 September). On this day, a decrease of 1.3 times in the maximum and the minimum temperatures and of 1.6 times in the relative humidity coincided with an important increase in *Alternaria spores* (4.8 times), *Pleosporales* spores (3.9 times) and Alt a 1 (7.1 times). This situation could not be explained by the observations on temperature reflected in the previous paragraph, although it agreed with those for relative humidity for the spores and for Alt a 1 in 2020. As a conclusion, longer datasets have to be obtained and analysed in order to better comprehend the relationship between spores and meteorology.

Despite there being a lot of studies about airborne allergens, these researches were focused on allergens from pollen and not from fungal spores. Most studies of aeroallergens from fungal spores were based on the detection and quantification of Alt a 1 [61,62,68,73] because it is the most important fungal aeroallergen that provokes respiratory tract symptoms. These studies were also helped by Elisa kits being available on the market for detection and quantification. The present study corroborates the results of these authors in the sense that airborne Alt a 1 can be detected and quantified using high-volume samplers and that its dynamics across the year are statistically related to the presence of airborne *Alternaria* spores.

Due to Horner et al. [1] describing that *Alternaria* had been shown to have very significant levels of allergenic cross reactivity with other fungi belonging to the Pleosporaceae family (*Curvularia*, *Stemphylium, Ulocladium*), that Saenz de Santamaría et al. [39] showed this, and that Rúça-Giraldo [45] cited the presence of Alt a 1 in other *Pleosporales*, in this study we performed a comparative analysis between Alt a 1 and spores of the order *Pleosporales* identified in the aerospore of Bellaterra and cited to contain Alt a 1: *Alternaria*, *Curvularia*, *Drechslera-Helminthosporium*, *Epicoccum*, *Lepthosphaeria*, *Pithomyces*, *Pleospora* and *Stemphylium*. The positive and significant correlation obtained between the two airborne spore taxa and Alt a 1 demonstrates that in the case of Alt a 1 sensitivity, it is important to know the levels of *Pleosporales* spores better than only those of *Alternaria*.

## 5. Conclusions

Alt a 1 daily concentration measurements (in alternate days) have been obtained, for the first time in Catalonia, at the Bellaterra (Barcelona) aerobiological station for the years 2019 and 2020. A MCV CAV-A/mb high-volume sampler (suction capacity of 400 L/min) has proven to be useful for the detection and quantification of the allergen Alt a 1 by means of Elisa kits. Two samplers located in close proximity are needed to obtain daily samples from 0 to 23:59 h. A significant correlation (*p* < 0.01) over 0.8 was found between airborne daily spore concentrations of the taxa *Alternaria* and *Pleosporales* (formed by *Alternaria*, *Curvularia*, *Drechslera-Helminthosporium*, *Epicoccum*, *Lepthosphaeria*, *Pithomyces*, *Pleospora* and *Stemphylium*); *Alternaria* spores accounted for more than 63% (and less than 67%) of the *Pleosporales* spores. Alt a 1 was better correlated with *Alternaria* spores than with *Pleosporales* spores. The results obtained show that to better help the diagnosis of Alt a 1 allergy and its treatment and emit alerts to prevent the exposition of the patients, the aerobiological monitoring is a good tool, especially if it can integrate the allergen and the two spores’ taxa considered in this study. As a minimum public health service, the study of the airborne *Alternaria* spores should be ensured.

## Figures and Tables

**Table 1:** Parameters studied for Alt a 1, *Alternaria* and *Pleosporales* spores obtained with the different samplers used, and meteorological data during 2017–2019–2020. ND: No Detection. __: no data.

		2017	2019	2020
Multi/vial Cyclone sampler	Alt a 1 AAIn (ng·day/m^3^)	ND	__	__
	Peak (ng/m^3^)	ND	__	__
	Peak day	ND	__	__
	Nr of days with Alt a 1	0	__	__
	Nr of analyzed days	359	__	__
High-volume sampler	Alt a 1 AAIn (ng·day/m^3^)	__	861.5	1352.0
	Peak (ng/m^3^)	__	30.6	109.7
	Peak day	__	29-Oct	25-Sep
	Nr of days with Alt a 1	__	155	173
	Nr of analyzed days	__	182	183
Hirst sampler	*Alternaria* spores ASIn (Spore·day/m^3^)	10,343	10,108	11,768
	Peak (spores/m^3^)	316	305	585
	Peak day	22-Oct	3-Nov	25-Sep
	Nr of days with *Alternaria* spores	335	326	316
	Analyzed days	361	363	339
	*Pleosporales spores* ASIn (Spore·day/m^3^)	16,285	15,733	17,657
	Peak (spores/m^3^)	356	372	658
	Peak day	22-Oct	3-Nov	25-Sep
	Nr of days with *Pleosporales* spores	357	356	335
	Nr of analyzed days	361	363	339
Meteorological data	TMax (°C)	21.7	21.6	21.5
	TMin (°C)	9.4	9.3	9.6
	TMean (°C)	15.6	15.5	15.5
	P 0–24 (mm)	468.8	553.0	847.2
	RH (%)	68.8	68.6	73.8

**Table 2:** Correlation coefficients for Alt a 1, *Alternaria* and *Pleosporales* spores and meteorological variables. ** *p* < 0.01; * *p* < 0.05. The shadowed and bold format indicate the more relevant results. N: number of days analyzed.

2017		Alt a 1	*Alternaria* spores	*Pleosporales* spores	TMax (°C)	TMin (°C)	TMean (°C)	P 0–24 (mm)	RH (%)
Alt a 1	Spearman Test								
	N	0	0	0	0	0	0	0	0
*Alternaria* spores	Spearman Test		1.000	0.860 **	0.639 **	0.601 **	0.631 **	−0.194 **	−0.224 **
	N	0	362	362	362	362	362	362	362
*Pleosporales* spores	Spearman Test		0.860 **	1.000	0.504 **	0.560 **	0.537 **	0.065	−0.092
	N	0	362	362	362	362	362	362	362
2019		Alt a	*Alternaria* spores	*Pleosporales* spores	TMax (°C)	TMin (°C)	TMean (°C)	P 0–24 (mm)	RH (%)
Alt a 1	Spearman Test	1.000	0.548 **	0.492 **	0.538 **	0.577 **	0.579 **	−0.043	−0.056
	N	155	153	155	155	155	155	155	155
*Alternaria* spores	Spearman Test	0.548 **	1.000	0.875 **	0.649 **	0.649 **	0.672 **	−0.077	−0.300 **
	N	153	362	362	362	362	362	362	362
*Pleosporales* spores	Spearman Test	0.492 **	0.875 **	1.000	0.541 **	0.619 **	0.599 **	0.163 *	−0.129 *
	N	155	362	362	362	362	362	362	362
2020		Alt a 1	*Alternaria* spores	*Pleosporales* spores	TMax (°C)	TMin (°C)	TMean (°C)	P 0–24 (mm)	RH (%)
Alt a 1	Spearman Test	1.000	0.631 **	0.556 **	0.566 **	0.550 **	0.571 **	−0.016	−0.229 *
	N	173	160	160	173	173	173	173	173
*Alternaria* spores	Spearman Test	0.631 **	1.000	0.856 **	0.614 **	0.556 **	0.602 **	−0.145 **	−0.326 **
	N	160	339	338	339	339	339	339	339
*Pleosporales* spores	Spearman Test	0.556 **	0.856 **	1.000	0.476 **	0.535 **	0.518 **	0.138 *	−0.119 *
	N	160	338	338	338	338	338	338	338

## Data Availability

The data presented in this study are available on request from the corresponding author. The data are not publicly available due to privacy.
